# Genomics in Lung Cancer: A Scoping Review of the Role of ctDNA in Non-Advanced Non-Small-Cell Lung Cancer in the Prediction of Prognosis After Multimodality Therapeutic Approaches

**DOI:** 10.3390/genes16080962

**Published:** 2025-08-15

**Authors:** Carolina Sassorossi, Jessica Evangelista, Alessio Stefani, Marco Chiappetta, Antonella Martino, Annalisa Campanella, Elisa De Paolis, Dania Nachira, Marzia Del Re, Francesco Guerrera, Luca Boldrini, Andrea Urbani, Stefano Margaritora, Angelo Minucci, Emilio Bria, Filippo Lococo

**Affiliations:** 1 Thoracic Surgery, Department of thoracic surgery, Fondazione Policlinico Universitario A. Gemelli Istituto di Ricovero e Cura a Carattere Scientifico (IRCCS), Università Cattolica del Sacro Cuore, 00168 Rome, Italy; carolina.sassorossi@guest.policlinicogemelli.it (C.S.); jessica.evangelista@policlinicogemelli.it (J.E.); marco.chiappetta@policlinicogemelli.it (M.C.); annalisa.campanella@guest.policlinicogemelli.it (A.C.); dania.nachira@policlinicogemelli.it (D.N.); stefano.margaritora@policlinicogemelli.it (S.M.); filippo.lococo@policlinicogemelli.it (F.L.); 2 Departmental Unit of Molecular and Genomic Diagnostics, Genomics Research Core Facility, Gemelli Science and Technology Park (GSTeP), Fondazione Policlinico Universitario A. Gemelli IRCCS, 00168 Rome, Italy; angelo.minucci@policlinicogemelli.it; 3 Medical Oncology, Department of Translational Medicine and Surgery, Università Cattolica del Sacro Cuore, 00168 Rome, Italy; alessio.stefani@unicatt.it; 4 Thoracic Surgery Unit, University “Magna Graecia”, 88100 Catanzaro, Italy; 5 Radiotherapy Unit, A. Gemelli University Hospital Foundation IRCCS, 00168 Rome, Italy; antonella.martino@policlinicogemelli.it; 6 Clinical Chemistry, Biochemistry and Molecular Biology Operations (UOC), Fondazione Policlinico Universitario A. Gemelli IRCCS, 00168 Rome, Italy; andrea.urbani@policlinicogemelli.it; 7 Thoracic Surgery Unit, Università Cattolica del Sacro Cuore, 00168 Rome, Italy; 8 Department of medical and health science, Saint Camillus International University of Medical and Health Sciences, 00128 Rome, Italy; marzia.delre@guest.policlinicogemelli.it; 9 Department of Cardio Thoracic and Vascular Surgery, Azienda Ospedaliera Universitaria Città della Salute e della Scienza di Torino, 10126 Turin, Italy; francesco.guerrera@unito.it; 10 Department of Radiology, Radiotherapy and Hematology, Fondazione Policlinico Universitario A. Gemelli IRCCS, 00168 Rome, Italy; luca.boldrini@policlinicogemelli.it; 11 Department of Basic Biotechnological Sciences, Intensivological and Perioperative Clinics, Catholic University of Sacred Heart, Largo Vito, 00168 Rome, Italy; 12 UOC Oncologia Medica, Isola Tiberina Gemelli Isola, 00186 Rome, Italy; emilio.bria@policlinicogemelli.it

**Keywords:** ctDNA, early stage diagnosis, NSCLC, surgery, radiotherapy, recurrence

## Abstract

**Background**: Circulating tumor DNA (ctDNA), shed into bodily fluids by cancer cells through apoptosis, necrosis, or active secretion, is currently used in the field of genomic investigation in clinical settings, primarily for advanced stages of non-small-cell lung cancer (NSCLC). However, its potential role in guiding the multi-omic approach to early-stage NSCLC is emerging as a promising area of investigation. Efforts are being made to integrate the genomics not only in surgery, but also in the definition of long-term prognosis after surgical or radiotherapy and for the prediction of recurrence. **Methods**: An extensive literature search was conducted on PubMed, covering publications from 2000 to 2024. Using the advanced search tool, titles and abstracts were filtered based on the following keywords: ctDNA, early stage, NSCLC. From this search, 20 studies that fulfilled all inclusion criteria were selected for analysis in this review. **Results**: This review highlights the growing body of evidence supporting the potential clinical use of ctDNA as a genomic biomarker in managing early-stage NSCLC. Baseline ctDNA levels offer valuable information about tumor molecular biology and histological characteristics. Beyond its prognostic value before treatment, liquid biopsy has proven useful for tracking minimal residual disease and forecasting recurrence following curative interventions such as surgery or radiotherapy. Future adjuvant treatment decisions may increasingly rely on predictive models that incorporate liquid biopsy findings alongside other clinical factors. **Conclusions**: The potential use of this analyte introduces new opportunities for the integration of genomic data in treatment, as well as relapse monitoring with more accurate and innovative than traditional methods, particularly in patients with early-stage NSCLC

## 1. Introduction

Defining the molecular profile of a tumor is crucial for enabling targeted cancer therapies and improving treatment success. Tumor-derived components found in blood and other bodily fluids can serve as valuable sources for genetic analysis. The most effective method for retrieving this genetic material from fluids is through what is known as a “liquid biopsy.” This approach addresses several limitations of tissue biopsies, such as challenges in obtaining surgical samples, and it offers the advantage of being repeatable, non-invasive, and well tolerated by patients, as it typically involves just a standard blood draw [1]. The concept of liquid biopsy emerged about a decade ago with the identification of circulating tumor cells and has since expanded to include other tumor-related elements such as circulating tumor DNA (ctDNA), circulating cell-free RNA, extracellular vesicles, and tumor-educated platelets [2].

ctDNA represents a subfraction of the cell-free DNA (cfDNA) specifically derived from tumor cells. These fragmented DNA molecules are shed following several cellular processes such as apoptosis, necrosis, or active secretion [3]. While cfDNA typically ranges in size from 100 to 800 base pairs, with a prominent fragment size around 160–180 base pairs corresponding to the length of DNA wrapped around a nucleosome [4], the ctDNA fragments are often shorter, typically 130–150 base pairs [4]. Increasing interest in ctDNA analysis has been observed in recent years, and different methodological approaches have been adopted for the evaluation of its translational value in oncology. A large portion of the existing studies focus on the quantitative estimation of the ctDNA rate obtained using methylation-based [5,6,7,8], sequencing-based [9,10], or fragmentomics-based [11,12] approaches. In fact, a more than 20-fold rise in cfDNA levels was observed in cancer-affected subjects with a ctDNA fraction ranging from 0.1–89%, which may increase with disease progression [13]. In addition to these approaches, the value of ctDNA relies on its ability to be a carrier of the same genetic background of the primary tumor and metastatic lesions. The actual clinical applications of liquid biopsy mainly consist in the ctDNA genotyping being a non-invasive source of molecular information about cancer [14,15,16]. These applications span from risk stratification to the monitoring of treatment response (i.e., molecular resistances and minimal residual disease) [14].

ctDNA utility as a prognostic biomarker has been demonstrated across multiple cancers, including breast, prostate, lung, and colorectal malignancies [17,18,19]. In 2022, the European Society for Medical Oncology (ESMO) issued recommendations supporting the use of ctDNA detection in plasma, stating that the available evidence justifies its routine clinical use to identify actionable genetic alterations [20]. Consequently, international oncology societies have begun to recommend incorporating ctDNA analysis into diagnostic workflows using next-generation sequencing (NGS) to evaluate tumor molecular profiles.

A particularly promising area of ctDNA application lies in managing early-stage non-small-cell lung cancer (NSCLC), both for baseline assessments and for detecting residual disease after curative interventions such as surgery or radiotherapy [21].

Lung cancer ranks among the most common and deadliest forms of cancer across the globe, representing the leading cause of cancer-related deaths. From a pathological perspective, it is typically divided into two major types: small-cell lung cancer (SCLC) and non-small-cell lung cancer (NSCLC). NSCLC accounts for about 85% of all lung cancer cases and includes subtypes such as lung adenocarcinoma (LUAD) and lung squamous cell carcinoma (LUSC), whereas the remaining 15% of cases are classified as SCLC. Recent statistics released by the International Agency for Research on Cancer (IARC) reveal that lung cancer represents approximately 12.4% of all new cancer diagnoses, and it is responsible for nearly 18.7% of all cancer-related fatalities. Among men, lung cancer has the highest incidence rate of all malignant tumors, while, among women, it ranks second after breast cancer [22].

As a key tool in the personalized treatment of lung cancer, especially in early-stage patients, ctDNA also enables ongoing monitoring to detect potential recurrence and guide the planning of adjuvant therapies. Its clinical use opens new opportunities for diagnosis, treatment, and follow-up that are more precise and less invasive than traditional methods, particularly for patients in the early stages of NSCLC.

The purpose of this narrative review is to highlight the most relevant clinical applications of ctDNA, especially its role in determining long-term prognosis following surgery or radiotherapy, as well as in predicting disease recurrence and patient outcomes.

## 2. Material and Methods

With the aim of analyzing studies of this issue, a literature review was performed.

An extensive literature review was conducted on PubMed, covering publications from 2000 to 2024. The process was independently performed by two reviewers (C.S. and J.E.), with oversight and conflict resolution provided by a third reviewer (F.L.). The advanced search function was utilized to screen titles and abstracts using the keywords: ctDNA, early stage, NSCLC, lung cancer, and circulating DNA.

Publication date range: studies published from 1 January 2000, through 30 November 2024, were considered. Language: only articles with full-text availability in English were included.

Study type: only peer-reviewed original research articles reporting primary data were eligible. Secondary sources such as reviews, editorials, letters, and other expert opinions were excluded during the initial screening. Conference abstracts and proceedings were also not considered. No restrictions were placed on the level of evidence.

Included studies focused on methods for detecting ctDNA, baseline ctDNA analysis for diagnosis, screening, histological characterization, pathological features, and staging of early-stage NSCLC. Additionally, studies assessing the role of ctDNA in predicting long-term prognosis post-surgery or radiotherapy, as well as in identifying disease recurrence, were incorporated.

Relevant data were extracted into a Microsoft Excel spreadsheet, including information on the author, study period, country, type of study, and the clinical purpose of ctDNA application. The final version of the manuscript was reviewed and approved by all co-authors, including the principal investigators of the included studies.

Exclusion criteria encompassed studies involving ctDNA in other tumor types or advanced-stage lung cancer, analyses focused on other blood-based biomarkers, and case reports, case series, and reviews. The full search strategy is outlined in Table 1.

### Literature Research Outcome

A detailed literature search on PubMed initially yielded 73 articles. Following a screening of titles and abstracts, studies classified as reviews, editorials, letters, commentaries, or case reports, along with three duplicates and non-English publications, were excluded. This resulted in 51 original articles deemed eligible and retrieved in full text, with no additional studies found through reference checking.

Of the full-text articles, 34 were excluded for various reasons: they focused on advanced-stage lung cancer or other tumor types, were written in languages other than English, or involved study populations of fewer than 10 patients. Ultimately, 17 studies fulfilled all inclusion criteria and were selected for inclusion in this review.

Concerning the excluded articles, some that were interesting but outside the scope of our review involved analyzing ctDNA to detect metastasis in early-stage lung cancer [23], or to predict the minimal residual disease as a parameter for tumor relapse [24]. Another very interesting study was about the use of ctDNA to predict the histology in non-biopsied advanced NSCLC candidates for stereotactic ablative radiotherapy [25]. Overall, the literature about this topic, using the cited keywords, is very rich in terms of the possible applications of ctDNA in clinical practice. Developing computational methods and bioengineering methodologies also play an emerging role in analyzing ctDNA data but, in this review, we took into account only the studies matching with our aims.

## 3. Results

### 3.1. Baseline ctDNA and Long-Term Prognosis After Minimally Invasive Surgery

In surgical settings, early-stage lung cancer is mainly treated with minimally invasive approaches, to enhance recovery after surgery. The presence of ctDNA after treatment can indicate residual microscopic disease, with persistent or rising levels postoperatively serving as an early marker of recurrence [26]. Similarly, pre-surgery ctDNA provides insight into tumor aggressiveness and can help to stratify patients for treatments [27]. In early-stage tumors, a significant decline or clearance of ctDNA after local treatment (both surgery or radiotherapy) typically correlates with better outcomes, while persistent levels may suggest treatment resistance or residual disease [26,27]. Recently published studies underscored the growing role of ctDNA as a powerful biomarker in early-stage NSCLC, particularly for the detection of the minimal residual disease (MRD) and for recurrence prediction.

Li et al. offered compelling evidence for ctDNA risk stratification and prognostic utility in their prospective study of perioperative ctDNA in 119 patients with stages I-IIIA resectable disease enrolled into GASTO1035-trial. By employing NGS on pre- and post-surgical plasma samples in a three-year timeframe, the authors observed that detectable ctDNA (both preoperative and within the initial postoperative phase) correlated with shorter relapse-free survival (RFS) and overall survival (OS). Remarkably, serial ctDNA detection with a tissue-informed approach identified recurrence almost nine months in advance of radiologic imaging, demonstrating its utility in the dynamic monitoring of the MRD. The study also underscored the importance of including ctDNA analysis in routine follow-up surveillance to facilitate timely interventions for recurrence [28].

Jung et al. focused on EGFR-mutant stage I-IIIA NSCLC in their longitudinal monitoring study on 278 patients. Their findings underscore the value of ctDNA as a marker for the early detection of recurrence and stratification of high-risk patients within a three-year timeframe. By utilizing droplet-digital PCR (ddPCR) for ctDNA analysis in a tissue agnostic approach at predefined intervals post-curative resection, the authors demonstrated its MRD detection and recurrence prediction abilities before clinical or radiologic signs emerged. The preoperative levels of ctDNA and its post-op clearance identified a group of patients with a high risk of recurrence after curative resection of disease. Additionally, the serial longitudinal monitoring of ctDNA proved its efficacy in detecting early recurrence prior to radiological confirmation (notably, 54% in exon-19 deletion and 11% in L858R-mutation cases), supporting its role as a non-invasive biomarker for recurrence surveillance [29].

In 2023, a pilot study by Markou et al. focused on the clinical importance of genetic mutations in patients with early-stage NSCLC. This study focused on the use of liquid biopsy components—specifically, plasma-derived cell-free DNA (cfDNA) and DNA from circulating tumor cells (CTCs)—prior to surgery. Researchers examined mutations in four key genes (*BRAF*, *EGFR*, *KRAS*, and *PIK3CA*) across three sample types: formalin-fixed paraffin-embedded tissue (FFPE), CTC-derived DNA, and plasma-cfDNA. A total of 49 operable NSCLC patients were included. Mutations were more frequently found in CTC-derived DNA (38.8%) compared to plasma-cfDNA (24.5%), which contrasts with some prior studies that reported higher mutation detection in plasma. This discrepancy may be attributed to the early disease stage in the study cohort. Among all genes analyzed, *PIK3CA* was the most commonly mutated, suggesting its possible role as a subclonal driver in early-stage NSCLC. Notably, patients who had no detectable mutations in either plasma-cfDNA or CTC-derived DNA showed significantly better relapse-free survival (RFS), highlighting the potential of liquid-biopsy-derived mutations as prognostic markers [30]. It has been suggested that CTCs and ctDNA can be used in a complementary manner in lung cancer studies. However, no clear large-scale confirmatory data are available and no concordance rate between these sources can be calculated [31]. In summary, some studies claim that CTC genotyping seemed to be more sensitive than ctDNA [32] and others state the opposite [33].

In another study, Peng et al. explored how ctDNA status—both before and after surgery—affects survival in 77 patients with resectable NSCLC. The results indicated that a negative ctDNA status prior to surgery and a lower stage of disease were both strong indicators of improved RFS and OS. Following curative surgery, ctDNA levels declined, especially in stage I and II patients, suggesting that early-stage tumors are less likely to leave residual disease after resection. The study emphasized the need for predictive biomarkers to better guide patient selection for adjuvant and neoadjuvant treatments, which are crucial for NSCLC in the early stage [34].

Xia et al. led a prospective observational study to assess the usefulness of ctDNA monitoring in lung cancer for evaluating post-surgical outcomes, tracking treatment response, and predicting relapse. The researchers examined 950 perioperative plasma samples from 330 patients with stage I–III NSCLC and found that the ctDNA-based detection of MRD was a more accurate predictor of RFS compared to conventional clinical factors. Preoperative ctDNA positivity was a strong prognostic marker, particularly in lung adenocarcinoma but less so in lung squamous cell carcinoma. Postoperative ctDNA positivity also indicated poorer RFS, suggesting that it reflects residual tumor cells. The study further demonstrated that MRD testing could guide adjuvant therapy decisions, with MRD-positive patients benefiting from such therapies, while MRD-negative patients showed worse outcomes with adjuvant treatments compared to MRD-negative patients not receiving any adjuvant treatment [35].

Another study by Waldeck and colleagues focused on ctDNA as a biomarker for relapse prediction. A total of 33 patients with stage IA to IIIB NSCLC scheduled for curative-intent surgery were prospectively enrolled. The study revealed that ctDNA was identifiable in 57% of the cases, with detection rates being notably higher in stage III patients compared to those in earlier stages. It highlighted that intraoperative manipulation of tumors can lead to increased ctDNA release, with ctDNA positivity detected one to two weeks after surgery, indicating a high risk of relapse. This study demonstrates that ctDNA-based monitoring could guide adjuvant therapies and spare patients without detectable ctDNA from unnecessary treatments. The study supports ctDNA as a non-invasive biomarker for relapse prediction, but limitations such as the small sample size are acknowledged [36].

Similar findings were produced by Tran and colleagues [16], who combined ctDNA analysis with CT imaging-based tumor volume to predict relapse risk in a subgroup of a cohort of 85 patients affected by resected stage I–III NSCLC. Their findings demonstrated that the detection of ctDNA before and after surgery correlate with worse clinical outcomes, and integrating ctDNA with tumor volume measurements enhanced the predictive accuracy for RFS and OS. This integrated approach confirms that the integrated framework of ctDNA and imaging may successfully support clinicians for predicting patients’ relapse risk and tailoring follow-up care [37].

Lastly, Tan et al. employed a personalized multiplex polymerase chain reaction (mPCR) NGS assay to longitudinally monitor ctDNA in 57 stage I–III NSCLC Asian patients. Their results suggested that presurgical ctDNA positivity was strongly related with shorter RFS, with ctDNA detection preceding recurrence on imaging by a median lead time of 2.8 months in 100% of the longitudinal post-surgical patients. This study underscores the clinical value of ctDNA in identifying MRD and guiding adaptive therapeutic and follow up strategies [38] (see the main findings reported in Table 2).

**Table 2:** Baseline ctDNA and long-term prognosis after surgery and radiotherapy.

First Author	Year	Country	Study Design	Approach Used for MRD	Result of ctDNA Detection	ctDNA Prognostic Impact	
						Pre-Treatment	Post-Treatment
Li [28]	2022	China	Prospective	Tissue informed	Post-operative serial ctDNA detection identified recurrence almost nine months earlier than conventional radiologic imaging	Yes	----
Jung [29]	2023	Korea	Retrospective	Tissue agnostic	ctDNA independent risk factor for FS ith stage (*p* < 0.001) and micropapillary subtype (*p* = 0.02). MRD detected before radiological recurrence in 69% of patients with exon 19 deletion and in 20% with L858R mutation.	Yes	----
Markou [30]	2023	Greece	Prospective	N/A	Univariate analysis showed that patients with detectable plasma-cfDNA mutations had a significantly increased risk of disease progression. This risk was even greater when mutations were present in either plasma-cfDNA or CTC-derived DNA. (HR: 2.716; 95% CI, 1.030–7.165; *p* = 0.043)	Yes	----
Peng [34]	2020	China	Prospective	Tissue informed	ctDNA-positive patients after surgery showed significantly lower RFS (HR = 3.076, *p* = 0.0015) and OS (HR = 3.195, *p* = 0.0053). Disease recurrence was observed in 63.3% (19/30) of ctDNA-positive patients, with 89.5% (17/19) of these patients showing detectable ctDNA within two weeks of surgery, on average 12.6 months before radiographic signs of recurrence.	----	Yes
Xia [35]	2022	China	Prospective	Tissue informed	The presence of ctDNA at either three days or one month after surgery was a powerful indicator of disease recurrence, showing a hazard ratio (HR) of 11.1 (*p* < 0.001).	----	Yes
Waldeck [36]	2022	Germany	Prospective	N/A	Detection of ctDNA in early postoperative plasma was linked to reduced progression-free survival (*p* = 0.013) and overall survival (*p* = 0.004).	----	Yes
Tran [37]	2024	USA	Prospective	N/A	Worse outcome for patients without ctDNA clearance after surgery.	----	Yes
Tan [38]	2024	Singapore	Retrospective	Tissue informed	ctDNA was identified in seven patients, all of whom later showed radiological evidence of recurrence. Notably, ctDNA positivity appeared before imaging confirmation, with a median lead time of 2.8 months (ranging from 0 to 12.9 months).	----	----
Walls [39]	2020	UK	Prospective	Tissue informed	ctDNA levels decreased during RT	----	----
Pan [40]	2023	China	Prospective	Tissue informed	ctDNA levels dropped significantly during chemoradiotherapy (CRT) at both the mid-treatment (on-RT) and post-treatment (after-RT) stages compared to baseline. A group of 38 patients (27.3%) who had undetectable ctDNA at both these time points—reflecting an early treatment response—demonstrated improved survival outcomes.	Yes	Yes

### 3.2. Baseline ctDNA and Long-Term Prognosis After Radiotherapy

**Figure 1:**
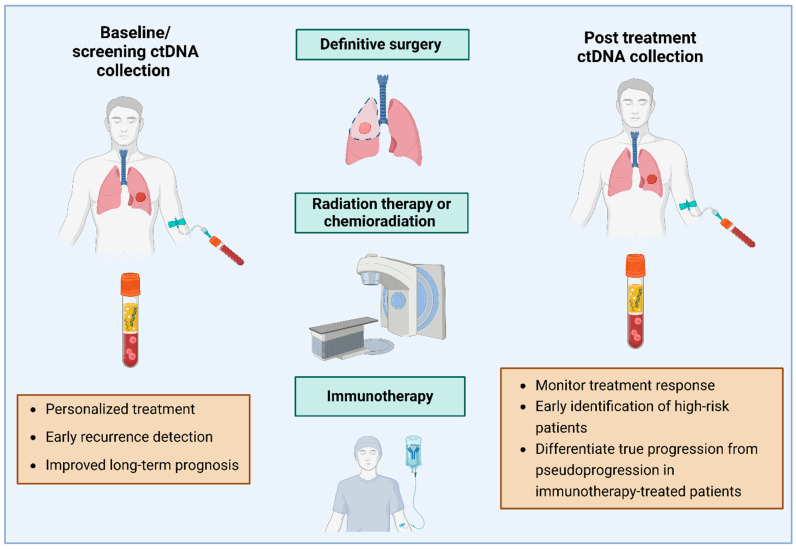
Integration of ctDNA and follow up after surgery, chemo- and radiotherapy and immunotherapy (generated using BioRender.com).

Although numerous clinical trials are ongoing to explore the role of ctDNA in the descripted areas, only a few focus on its use in patients undergoing radiation treatment (RT) alone or in combination with chemotherapy or immunotherapy [1] (Figure 1).

Walls et al. [39] explored the use of ctDNA in locally advanced NSCLC patients undergoing RT in 2020. Their study demonstrated that ctDNA levels decreased during RT, possibly due to reduced tumor cell presence or impaired DNA release. Plasma-detectable mutations showed a reduction by day seven in all patients. However, two individuals exhibited a temporary rise in ctDNA levels at the 72 h mark compared to baseline. On average, ctDNA levels slightly increased at 72 h and then declined by day seven relative to the baseline. Nonetheless, these changes were not statistically significant. The pilot study confirmed the feasibility of ctDNA monitoring during radiotherapy, although more research is needed to optimize timing and improve sensitivity, as well as to differentiate between tumor-related mutations and other genetic variations.

ctDNA has been revealed to be a valuable prognostic biomarker for MRD assessment and recurrence prediction. Lebow et al. [41] conducted a study highlighting the potential of tumor-informed ctDNA monitoring as an effective method for detecting MRD in patients with localized lung cancer after receiving definitive radiation therapy (RT). In this prospective study, 70 plasma samples were sequentially collected from 17 NSCLC patients at various points—before, during, and after treatment. The findings revealed that patients who tested positive for ctDNA at the first post-RT timepoint had significantly shorter progression-free survival (PFS) compared to ctDNA-negative patients (hazard ratio [HR]: 24.2, 95% confidence interval (CI): 2.8–208.6, *p* = 0.004). This correlation between ctDNA positivity and reduced PFS was further validated by additional analyses (HR: 24.2, *p* = 0.004 and HR: 13.4, *p* = 0.02). ctDNA monitoring can detect recurrences months before clinical symptoms, offering an important window for early intervention. In early-stage cancer, ctDNA could help in the identification of patients who would benefit from adjuvant treatment. In the context of locally advanced disease, ctDNA may also help personalize treatment, such as by guiding the use of immunotherapy.

With a similar aim, Pan et al. [40] conducted the largest prospective study to date in 2023, investigating ctDNA-based MRD detection in NSCLC patients treated with definitive radiotherapy. The study examined 761 peripheral blood samples collected from 139 individuals with unresectable, locally advanced NSCLC undergoing definitive RT. Among these, 17.7% of patients achieved undetectable MRD levels, and the majority of them remained free from disease progression throughout the follow-up period. ctDNA-MRD detection may represent a powerful tool for predicting patient outcomes, guiding treatment, and potentially avoiding overtreatment (main findings reported in Table 2).

### 3.3. Post-Treatment ctDNA and Prediction of Recurrence and Prognosis

Beyond its prognostic significance before treatment, liquid biopsy has proven to be a valuable approach for tracking residual disease and anticipating recurrence in early-stage NSCLC following curative therapies. Detecting ctDNA in the postoperative phase provides a distinct opportunity to identify MRD, offering important prognostic insights regarding relapse risk and overall outcomes. The prognostic impact of MRD was evaluated in a meta-analysis by Liang et al., which examined studies assessing both ctDNA and CTCs in the postoperative context. This analysis, which included 351 patients, revealed that the postoperative presence of either ctDNA or CTCs was linked to significantly worse disease-free survival (DFS) (ctDNA HR: 8.15, 95% CI: 2.11–31.50, *p* = 0.002; CTCs HR: 3.37, 95% CI: 2.28–4.96; *p* < 0.001) [42].

The timing of sample collection to identify MRD varies across studies, ranging from a few days to a few months. ctDNA has a half-life of two to three hours in serum [34,35,43,44,45], leading to its rapid clearance after a curative approach [46]. For this reason, it could be evaluated shortly after the treatment. Two different strategies have been studied to predict the risk of recurrence: the landmark strategy, i.e., a single sample collected within the first one to four months after definitive treatment, and the surveillance strategy, i.e., longitudinal ctDNA monitoring during the follow-up phase. A recent meta-analysis compared the reliability of these strategies, concluding that, while specificity is high for both (slightly higher in the case of the landmark strategy), sensitivity is disappointingly low, although it could be enhanced with the longitudinal monitoring of the surveillance strategy [47]. The latter has the obvious disadvantage of higher costs, making it more difficult to fit in the clinical practice.

There are two primary strategies for detecting ctDNA-based minimal residual disease (MRD): the tumor-informed approach, which relies on sequencing the original tumor tissue to identify specific mutations for personalized ctDNA tracking; and the tumor-agnostic method, which utilizes predefined panels to detect common genomic or epigenomic alterations in plasma without requiring prior tumor analysis. In a prospective study involving 29 early-stage NSCLC patients who underwent surgical resection, post-operative samples were assessed using a tumor-informed targeted NGS assay. Liquid biopsy was conducted alongside CT imaging, and ctDNA was identified in 50% of the patients who showed disease recurrence on imaging (two out of four cases).

However, the technique used was insufficiently sensitive to reliably detect MRD, limiting its utility to confirming the presence of disease in cases of ambiguous imaging and ctDNA positivity. Furthermore, the two patients with disease recurrence but negative liquid biopsy results had undergone stereotactic RT at the recurrence sites prior to blood sample collection, which may have influenced the findings [41]. Another tumor-informed NGS assay was used in a retrospective study involving 36 patients, 24 of whom had blood samples collected within the MRD window (defined as within six months post-surgery). Detectable ctDNA was identified in two patients, who exhibited a 15-fold higher probability of recurrence of disease if compared to those with negative ctDNA (HR for DFS 15.0, 95% CI: 1.0–253.0, *p* = 0.010). A positive liquid biopsy during the follow-up phase (nine patients) also correlated with worse DFS compared to patients with persistently negative ctDNA results (*p* < 0.0001) [48]. Martin et al. led a prospective study on a cohort of 108 NSCLC patients who underwent longitudinal collection of blood samples after surgery. ctDNA was analyzed using a tumor-informed assay. Although the follow-up was limited, 11% of patients were ctDNA positive after surgery and post-operative clinical management was altered in all of them, anticipating radiological imaging. A PET scan confirmed disease recurrence in 8 of those 12 ctDNA+ patients (66%). Of patients with radiological recurrence (*n* = 10), eight had ctDNA detectable by liquid biopsy while two of them had only CNS disease. Comparing the risk of recurrence in patients based on ctDNA status, patients who were ctDNA positive had a significantly worse DFS (HR = 27, 95%CI: 5.6–127, *p* < 0.0001) [49].

Qiu et al. used a targeted NGS-panel to detect MRD in a cohort of NSCLC patients after surgical treatment, after adjuvant treatment, and during the follow-up. In total, 18 of 85 patients (21.2%) who had a post-surgical sample available resulted ctDNA+. The risk of relapse was significantly higher for these patients (HR:4.0; 95% CI: 2.0–8.0; *p* < 0.001). Moreover, 64 patients completed adjuvant therapy and 8 of them were ctDNA+; again, the risk of relapse was higher for those patients (HR, 3.2; 95% CI, 1.3–8.2; *p* < 0.05). The researchers also evaluated the impact of adjuvant chemotherapy (ACT) based on postoperative ctDNA status. Among ctDNA-negative patients, the risk of relapse remained low regardless of ACT administration (*p* = 0.46). In contrast, ctDNA-positive patients who received ACT experienced significantly improved relapse-free survival (RFS) compared to those who did not undergo ACT (*p* < 0.05) [44].

Concerning oncogene-addicted disease, a prospective study conducted by Jung et al. was conducted on 278 patients with early-stage *EGFR*-mutated NSCLC. Samples for ctDNA detection were obtained both before and after surgery, enabling the stratification of patients into three cohorts: those who were ctDNA negative both pre- and post-surgery, those who were ctDNA positive before surgery but became negative after resection, and those who remained ctDNA-positive both before and after surgery. The three-year DFS was, respectively, 84%, 78% and 50% (*p* = 0.02). In this analysis, the researchers used ddPCR to identify *EGFR* sequences in ctDNA. Molecular progression anticipated radiological recurrence in 69% of exon 19-delection-cases and 20% of L858R-cases [29].

Gale et al. conducted a prospective study of 88 patients with NSCLC who were surgically treated or treated with definitive radiotherapy. In the follow-up period, 28 patients experienced disease progression and 18 of them were ctDNA+ (64.3%). Positivity of ctDNA at a landmark timepoint (from two weeks to four months following curative treatment) was related to a worse prognosis (HR 5.48, *p* = 0.00029 for OS; HR 14.8, *p* < 0.0001 for DFS), and the average time between the detection of ctDNA and the clinical confirmation of disease recurrence was approximately seven months [50]. Similarly, another prospective study conducted on 17 patients treated with definitive radiotherapy concluded that ctDNA could anticipate radiological progression of about five months [41] (the main findings are reported in Table 3).

### 3.4. Future Directions

Looking ahead, ctDNA has the potential to become a non-invasive, cost-effective, and efficient tool in routine clinical practice for monitoring treatment response after definitive therapies, identifying patients at high risk of relapse, and distinguishing true disease progression from pseudo-progression. In a study utilizing data from the Phase 3 IMpower150 trial, Ding et al. explored the prognostic value of ctDNA metrics in NSCLC patients undergoing first-line chemoimmunotherapy. Their findings revealed a strong correlation between post-treatment ctDNA dynamics and imaging responses, emphasizing the significance of ctDNA nadir levels in predicting both PFS and OS. The authors suggested that combining ctDNA analysis with radiological evaluations, particularly during weeks six to nine, could improve the prediction of long-term outcomes [51].

The introduction of immunotherapy in the neoadjuvant and/or adjuvant phase has recently changed the landscape of treatment of NSCLC. In the IMpower-010 trial, which established the role of adjuvant atezolizumab after ACT, 600 of 1005 enrolled patients who underwent a liquid biopsy to assess the presence of ctDNA after surgery. DFS was significantly worse in the 21% of patients that resulted ctDNA+, although. However, both ctDNA+ and ctDNA- patients achieved better outcomes with adjuvant atezolizumab compared to best supportive care. Therefore, the non-invasive detection of ctDNA after adjuvant treatments can also identify patients at risk of disease recurrence before radiological imaging allows nowadays.

A clinical trial currently in progress (NCT04966663) is investigating whether patients with resected stage I NSCLC might benefit from intensified treatment if ctDNA remains detectable following surgery.

**Figure 2:**
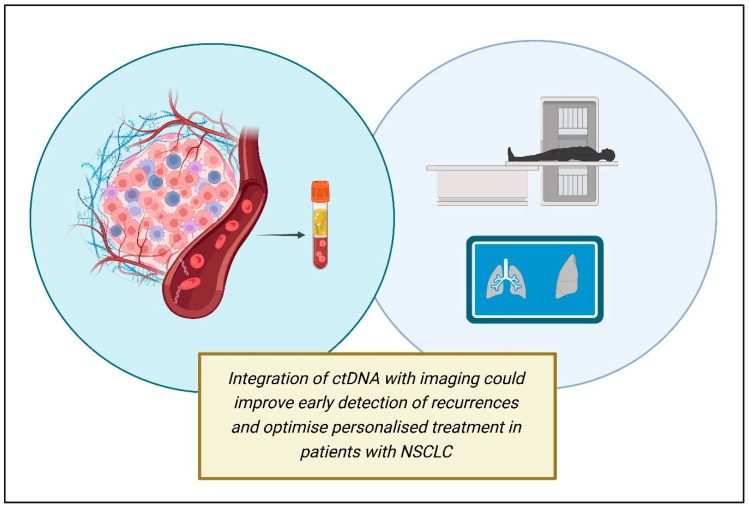
Future directions in ctDNA applications (created using BioRender.com).

ctDNA also could represent a dynamic predictive biomarker to help distinguish pseudoprogression under immune check-point inhibitor (ICI) therapy from real progression [52]. Indeed, pseudo-progression (defined as the radiological finding of disease, caused by immune cells infiltrating the tumor mass) is a diagnostic challenge in ICI treatments. Therefore, the monitoring of early ctDNA variations and changes in concentration during immunotherapy can better predict the efficacy of therapy, guiding the following clinical decisions (Figure 2).

## 4. Discussion

The findings discussed above indicate the potential for incorporating ctDNA analysis into routine clinical workflows—not only for predicting recurrence and long-term outcomes [27], but also in contexts where precision medicine is essential for patient stratification, identifying intrinsic resistance mechanisms, optimizing treatment strategies, and guiding overall disease management [26,38,52].

The utility of ctDNA has emerged as a transformative tool for early identification, management, and surveillance in early-stage NSCLC. As highlighted in our review, ctDNA analysis offers a minimally invasive chance, enabling real-time molecular profiling, disease control, and recurrence prediction.

Abbosh and colleagues [3] identified factors associated with ctDNA detectability in early-stage NSCLC, including non-adenocarcinoma histology, tumor necrosis, high proliferation rates, and lymphovascular invasion. For instance, a primary NSCLC tumor volume of 10 cm^3^ was predictive of a ctDNA plasma variant allele frequency (VAF) of 0.1%.

The detection of ctDNA after definitive treatment clearly identifies a group of patients at higher risk for disease recurrence and may even predict radiological relapse, providing a valuable opportunity for early intervention. Indeed, recent advancements in next-generation sequencing technologies and targeted assays have significantly improved the sensitivity and specificity of ctDNA detection, particularly in identifying MRD and predicting relapse. However, the variability in detection rates across histological subtypes and stages underscores the need to integrate ctDNA analysis with imaging and other biomarkers to optimize diagnostic and prognostic accuracy. In particular, one of the major challenges for ctDNA detection especially in lung cancer includes ctDNA shedding. ctDNA is strongly influenced by the biological characteristics of the originating tumor: the tumor burden, the localization, the proliferating index or the ground glass opacity all influence the ctDNA shedding [53,54,55]. Therefore, ctDNA shedding may strongly challenge a clear identification of true negatives versus false negatives, influencing the evaluation and the stratification of patients ctDNA positive versus negatives. Ongoing progress is crucial to confirm the utility of liquid biopsy as a means of informing treatment choices in the adjuvant context, with the promise of tailoring therapy according to the molecular evolution of the disease.

## 5. Conclusions

In summary, upcoming research should aim to overcome existing challenges, including the need to standardize both pre-analytical and analytical protocols, as well as to validate ctDNA testing through large-scale prospective clinical studies. Incorporating ctDNA into comprehensive diagnostic approaches has significant potential for advancing personalized treatment plans and enhancing outcomes for patients with early-stage NSCLC.

## Figures and Tables

**Table 1:** Search strategy summary.

Search Date	10 December 2024
Databases searched	Pubmed
Keywords used	“ctDNA” and “early stage” and “NSCLC”
Time period	1 January 2000 to 30 November 2024
Eligibility criteria	Only peer reviewed: clinical trial (randomized, prospective, or retrospective) or original article, only written in English
Screening process	The abstracts found through this search were independently examined by two authors (C.S. and M.C.), and in case of any disagreements, a third author (F.L.) was consulted for resolution.

**Table 3:** ctDNA in recurrence prediction: main findings.

First Author	Year	Country	Study Design	Outcome in ctDNA Detection
Gale [50]	2022	UK	Prospective	The median time between ctDNA detection and clinically confirmed recurrence was approximately seven months. ctDNA positivity at a predefined landmark timepoint (ranging from two weeks to four months following curative treatment) was associated with poorer prognosis. (HR 5.48, *p* = 0.00029 for OS; HR 14.8, *p* < 0.0001 for DFS)
Jung [29]	2023	Korea	Prospective	Three-year DFS ctDNA-positive pre-surgery negative, after resection 84%, ctDNA-positive pre-surgery and remained positive at 78%
Liang [42]	2019	China	Prospective	ctDNA or CTCs after surgery associated with poorer DFS (ctDNA HR = 8.15, 95%CI = 2.11–31.50, *p* = 0.002; CTCs HR = 3.37, 95%CI = 2.28–4.96; *p* < 0.001
Qiu [44]	2021	China	Prospective	After surgery, 18 of 85 patients (21.2%) had ctDNA+, with significantly higher risk of relapse (HR = 4.0; 95%CI = 2.0–8.0; *p* < 0.001). After adjuvant therapy, 8 out of 64 patients had ctDNA+; risk of relapse was higher for those patients (HR = 3.2; 95%CI, 1.3–8.2; *p* < 0.05)
Naso [48]	2024	Canada	Prospective	Here, 2 out of 24 patients were ctDNA+ within nine months after treatment and had a 15-fold higher probability of recurrence compared to those with negative ct-DNA (HR for DFS 15.0, 95% CI: 1.0–253.0, *p* = 0.010
Oh [49]	2024	USA	Prospective	Patients with ctDNA+ had a significantly worse DFS (HR = 27, 95%CI = 5.6–127, *p* < 0.0001)
Lebow [41]	2023	USA	Prospective	ctDNA could anticipate radiological progression of about five months

## Data Availability

Data sharing is not applicable to this article as no datasets were generated or analyzed during the current study.
